# Predicting Prolonged Apnea During Nurse-Administered Procedural Sedation: Machine Learning Study

**DOI:** 10.2196/29200

**Published:** 2021-10-05

**Authors:** Aaron Conway, Carla R Jungquist, Kristina Chang, Navpreet Kamboj, Joanna Sutherland, Sebastian Mafeld, Matteo Parotto

**Affiliations:** 1 Lawrence S. Bloomberg Faculty of Nursing University of Toronto Toronto, ON Canada; 2 Peter Munk Cardiac Centre Toronto General Hospital Toronto, ON Canada; 3 School of Nursing Queensland University of Technology Brisbane Australia; 4 School of Nursing The University at Buffalo Buffalo, NY United States; 5 Rural Clinical School University of New South Wales Coffs Harbour Australia; 6 Joint Department of Medical Imaging Toronto General Hospital Toronto, ON Canada; 7 Department of Anesthesia and Pain Management Toronto General Hospital Toronto, ON Canada; 8 Department of Anesthesiology and Pain Medicine University of Toronto Toronto, ON Canada; 9 Interdepartmental Division of Critical Care Medicine University of Toronto Toronto, ON Canada

**Keywords:** procedural sedation and analgesia, conscious sedation, nursing, informatics, patient safety, machine learning, capnography, anesthesia, anaesthesia, medical informatics, sleep apnea, apnea, apnoea, sedation

## Abstract

**Background:**

Capnography is commonly used for nurse-administered procedural sedation. Distinguishing between capnography waveform abnormalities that signal the need for clinical intervention for an event and those that do not indicate the need for intervention is essential for the successful implementation of this technology into practice. It is possible that capnography alarm management may be improved by using machine learning to create a “smart alarm” that can alert clinicians to apneic events that are predicted to be prolonged.

**Objective:**

To determine the accuracy of machine learning models for predicting at the 15-second time point if apnea will be prolonged (ie, apnea that persists for >30 seconds).

**Methods:**

A secondary analysis of an observational study was conducted. We selected several candidate models to evaluate, including a random forest model, generalized linear model (logistic regression), least absolute shrinkage and selection operator regression, ridge regression, and the XGBoost model. Out-of-sample accuracy of the models was calculated using 10-fold cross-validation. The net benefit decision analytic measure was used to assist with deciding whether using the models in practice would lead to better outcomes on average than using the current default capnography alarm management strategies. The default strategies are the aggressive approach, in which an alarm is triggered after brief periods of apnea (typically 15 seconds) and the conservative approach, in which an alarm is triggered for only prolonged periods of apnea (typically >30 seconds).

**Results:**

A total of 384 apneic events longer than 15 seconds were observed in 61 of the 102 patients (59.8%) who participated in the observational study. Nearly half of the apneic events (180/384, 46.9%) were prolonged. The random forest model performed the best in terms of discrimination (area under the receiver operating characteristic curve 0.66) and calibration. The net benefit associated with the random forest model exceeded that associated with the aggressive strategy but was lower than that associated with the conservative strategy.

**Conclusions:**

Decision curve analysis indicated that using a random forest model would lead to a better outcome for capnography alarm management than using an aggressive strategy in which alarms are triggered after 15 seconds of apnea. The model would not be superior to the conservative strategy in which alarms are only triggered after 30 seconds.

## Introduction

With the recent increase in the use of electronic monitoring devices in the hospital setting, alarm fatigue has become a serious problem that impacts patient safety and nursing care [1]. Alarm fatigue is caused by exposure to excessive and frequent device alarms and leads to desensitization to alarms. Alarm fatigue has been linked to patient deaths resulting from delayed responses to clinical deterioration by clinicians who have become desensitized to alarms [2]. One of the sources of alarms is the capnography device that is used to measure and monitor ventilation in patients. A capnography waveform displays the level of expired carbon dioxide (CO_2_) over time to show changes in concentrations throughout the respiratory cycle. Capnography waveform abnormalities assist in detecting and diagnosing specific conditions such as partial airway obstruction and apnea. For this reason, the implementation of capnography into practice for respiratory monitoring is considered a high priority to improve patient safety by leading authorities, including national and international professional organizations for anesthesiology in Canada, the United States, and Europe [3-5]. Capnography is commonly used for nurse-administered procedural sedation [6-8], including in the interventional radiology setting [9-13].

Distinguishing between the capnography waveform abnormalities that signal the need for clinical intervention for an event and those waveform abnormalities that do not indicate the need for intervention is essential to the successful implementation of this technology into practice. For example, alarms triggered after short periods of apnea lead to frequent interruptions and potentially increase the risk of alarm fatigue. Conversely, intervention provided only when an apneic event reaches a longer threshold negates the potential benefits that capnography can have on patient safety through improved ventilation. In practice, two alternative strategies for capnography alarm management are typically used. The aggressive strategy involves alarms triggered after short periods of apnea (typically 15 seconds). The conservative approach involves alarms triggered only when an apneic event is prolonged (typically >30 seconds). Preferences for the aggressive or conservative alarm threshold are influenced by many factors, including the rate of oxygen supplementation. The duration of time between the onset of apnea to hypoxemia increases with higher oxygen flow [14].

Capnography alarm management may be improved by using machine learning to create a “smart alarm” that can alert clinicians to apneic events that are predicted to be prolonged. Such an approach aligns with a call from The Society for Critical Care Medicine Alarm and Alert Fatigue Task Force that machine learning techniques should be used to advance the quality of alerts that clinicians receive and to individualize alert delivery based on clinician response characteristics such as alert frequency and event severity [15].

In the aggressive alarm management strategy, if an alarm is only triggered for apneic events predicted to be prolonged, it would reduce the total alarm burden and potentially reduce the risk of alarm fatigue. The downside of applying a machine learning approach to the aggressive strategy would be that some patients with prolonged apnea may not receive early intervention if the model incorrectly predicts that the apneic event will not last for >30 seconds (ie, false negatives). In the conservative alarm management strategy, if an alarm is triggered at the 15-second timepoint for apneic events predicted to be prolonged, it could reduce the total time of the apneic event because treatment could be initiated earlier. The downside of applying a machine learning approach to the conservative strategy would be the potential increase in the total alarm burden if the ratio of false positives (apnea incorrectly predicted to last for >30 seconds) to true positives (apnea correctly predicted at the 15-second time point to last for >30 seconds) is high. This study aimed to determine the accuracy of machine learning models for predicting at the 15-second timepoint if an apneic event will persist for >30 seconds. This information would help determine whether operationalizing these predictions into practice as alarm triggers would be beneficial.

## Methods

We performed a secondary analysis of a prospective observational study. The primary aim of the observational study was to identify common patterns in capnography waveform abnormalities and factors that influence these patterns [16]. All participants provided written informed consent and the study was approved by human research ethics committees (UCH HREC 1614; SVHAC HREC 16/26; QUT 1600000641).

### Prediction Goal

The prediction goal was to classify apneic events at the 15-second timepoint as either short (ie, terminated before 30 seconds) or prolonged (persisted for >30 seconds). The prediction algorithm was compared against typical default alarm settings for capnography monitors.

### Participants

Participants in the observational study were consecutive adult patients who were scheduled to undergo an elective procedure in the cardiac catheterization laboratory with moderate sedation. Patients with severe cognitive impairment who could not provide informed consent and those unable to understand and speak English (in the absence of an interpreter) were excluded. Data collection was performed at two urban private hospitals in Australia.

### Sedation and Monitoring

The sedation regimen used for patients included in this study comprised bolus doses of intravenous midazolam and fentanyl. Sedation was performed by nurses who were trained in advanced life support. Routine clinical monitoring included continuous cardiac rhythm and oxygen saturation monitoring as well as noninvasive blood pressure measurements every 5-10 minutes. The Respironics LoFlo Sidestream etCO_2_ sensor was used for capnography monitoring. A CO_2_ sampling cannula was inserted into the side port of an oxygen face mask or was integrated as a separate line for nasal cannulas. The capnography waveform was displayed on the main physiological monitoring screen. A default “No breaths detected” alert was triggered for apnea, but no other audible or visual alarms were set for the capnography monitor. No restrictions or specific instructions regarding the detection of capnography waveform abnormalities were provided to clinicians as part of the research protocol because the study used an observational design.

### Data Collection

Data were collected from August 2016 to May 2018. Demographic data and clinical characteristics were collected from medical records or directly from participants prior to procedures. Intraprocedural data were collected in real time by the researcher present in the procedure room. Direct observation of the participant was required to record the timing of sedation administration and any interventions by sedation providers.

### Predictor Variables

Several raw demographic (age, sex) and clinical (American Society of Anesthesiology physical status classification, diagnosis of sleep apnea, BMI, dose and type of sedative and analgesic administered) variables were used as predictors. Features related to sedation regimen dosing used as predictors in the model were the total sedative dose and number of sedative doses administered, time since first sedation, and time since the previous sedative dose. Other features were extracted from the capnography waveform for use as predictors, such as the previous respiratory state (normal or abnormal breathing), duration of the previous apneic event, time since the previous apneic event, and total number of apneic events. A total of 18 predictor variables were used.

### Statistical Analysis

Analyses were performed using R version 4.0.3 [17]. Data as well as details about how to access the code and a reproducible computing environment to verify the results were available [18,19].

### Modeling

We selected several candidate models to evaluate, including a random forest model, generalized linear model (logistic regression), least absolute shrinkage and selection operator regression, ridge regression, and the XGBoost model. Out-of-sample accuracy of the models was calculated using 10-fold cross-validation. Many participants in the study contributed multiple apneic events to the dataset used for modeling. To take this dependency into account, we ensured that apneic events from individual participants were not included in both the training and testing partitions of the 10-fold cross-validation process. Preprocessing steps included normalizing numeric predictors and using an interaction term for the duration of the previous respiratory state and the total number of apneic events. The discriminatory ability of the models was compared using the area under the receiver operating characteristic curve (AUROC) as well as by plotting sensitivity, specificity, positive predictive values, and negative predictive values (termed a threshold performance plot). A calibration plot with locally estimated scatterplot smoothing was used to assess calibration [20]. The runway package was used to create the plots [21].

### Decision Curve Analysis

We used the net benefit decision analytic measure to assist with deciding whether using the models in practice would lead to better outcomes on average than using the current default capnography alarm management strategies. The default strategies are the aggressive approach, in which an alarm is triggered after brief apneic events (typically 15 seconds), and the conservative approach, in which an alarm is triggered for only prolonged apneic events (typically >30 seconds). Calculation of the net benefit essentially transforms the total number of true positives (apneic event predicted to be prolonged at 15 seconds and correctly persisted for >30 seconds) and false positives (apneic events predicted to be prolonged at 15 seconds but did not persist for >30 seconds) into a standardized scale, weighted by the relative harm of a false-positive result [22]. For example, a net benefit of 0.07 means that the net benefit of using the model would be 7 true positives from every 100 predictions from the model. This net benefit can result from any combination of true positives and false positives [23]. A probability threshold of 0.5 indicates that avoiding a false positive is as important to a clinician as identifying a true positive. Preferences for probability thresholds below 0.5 are weighted such that identifying a true positive is more valuable than avoiding a false positive. Preferences for probability thresholds above 0.5 are weighted such that avoiding a false positive is more valuable than identifying a true positive. For example, for a probability threshold of 0.75, the value of a false positive is 3 true positives (0.75/0.25). In other words, to create a net benefit from using the model at this probability threshold, there must be more than 3 true positives for every false positive prediction made from the model. Conversely, for a probability threshold of 0.25, the value of a false positive is weighted far lower, at only one-third of a true positive (0.25/0.75). This means that a net benefit would be achieved if there were more than 1 true positive for every 3 false positives. Decision curves can be interpreted such that the strategy with the highest net benefit at each probability threshold has the highest clinical value [23].

We created a decision curve to plot net benefits across a range of probability thresholds for the aggressive strategy (alarm triggered at 15 seconds of apnea) and the conservative alarm management strategy (alarm triggered at 30 seconds of apnea). The decision curve takes into account the full range of reasonable clinician preferences for the point at which an alarm should be triggered to signal an apneic event in a patient. We tested thresholds in the range of 0.3-0.5 for the aggressive strategy. In the practical sense, this means that we decided that all clinicians who usually use the aggressive strategy would not accept a probability of prolonged apnea lower than 0.3 as a useful alarm trigger because there would be little difference between this strategy and simply setting the alarm for all apneic events. We also decided that all clinicians would always consider that an alarm is triggered for the aggressive strategy if the probability of prolonged apnea was higher than 0.5. A range of values was used because these probability thresholds can be interpreted as value preferences that individual clinicians may reasonably choose in clinical practice. For example, a clinician who is more risk-averse may select a more conservative probability threshold (closer to 0.3). Individual participant characteristics will also influence clinicians’ decisions about probability thresholds. A clinician may elect to intervene when the probability of prolonged apnea is 0.3 for an older patient with multiple comorbidities but not for a young patient who may more likely be able to tolerate longer periods of apnea. For the conservative strategy, we chose to plot the range of probability thresholds from 0.7 to 0.8. Higher values were chosen because the number of false positives would be an important consideration for clinicians already using a conservative alarm management approach.

## Results

A total of 384 apneic events of at least 15 seconds duration from 61 of the 102 patients (59.8%) who participated in this observational study were included in the present analysis. A summary of participant characteristics is presented in Table 1. Nearly half of the apneic events (180/384, 46.9%) were prolonged (ie, >30 seconds).

**Table 1:** Participant characteristics (N=61).

Characteristic			Value
Age, median (IQR)			76 (68-80)
Body mass index, median (IQR)			26.4 (24.6-29.8)
**Sex, n (%)**			
	Female	22 (36)	
	Male	39 (64)	
**Obstructive sleep apnea, n (%)**			
	No	46 (75)	
	Yes	15 (25)	
**American Society of Anesthesiology physical status** **classification, n (%)**			
	I or II	37 (61)	
	III or IV	24 (39)	

### Discrimination

**Figure 1:**
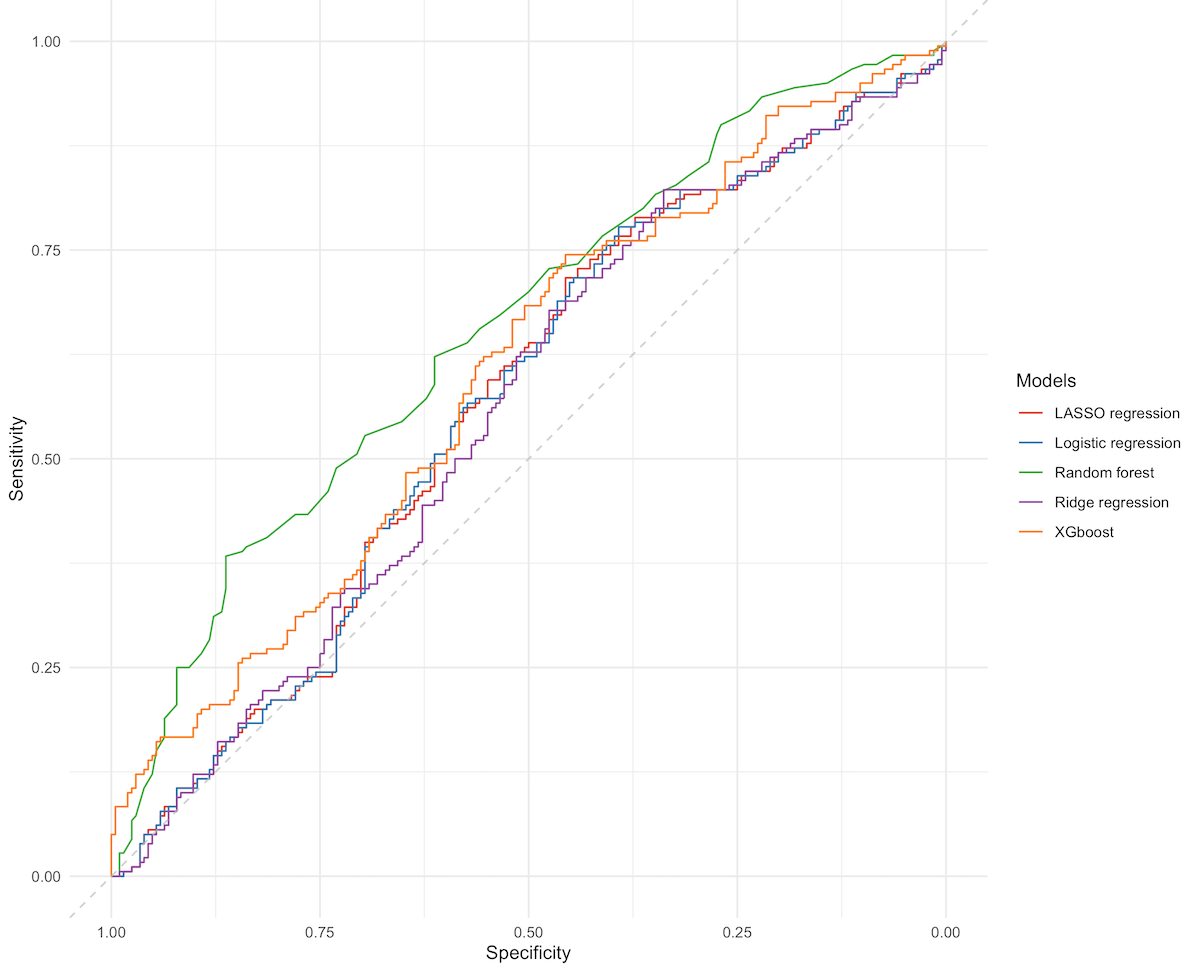
Area under the receiver operating characteristics curve. LASSO, least absolute shrinkage and selection operator.

**Figure 2:**
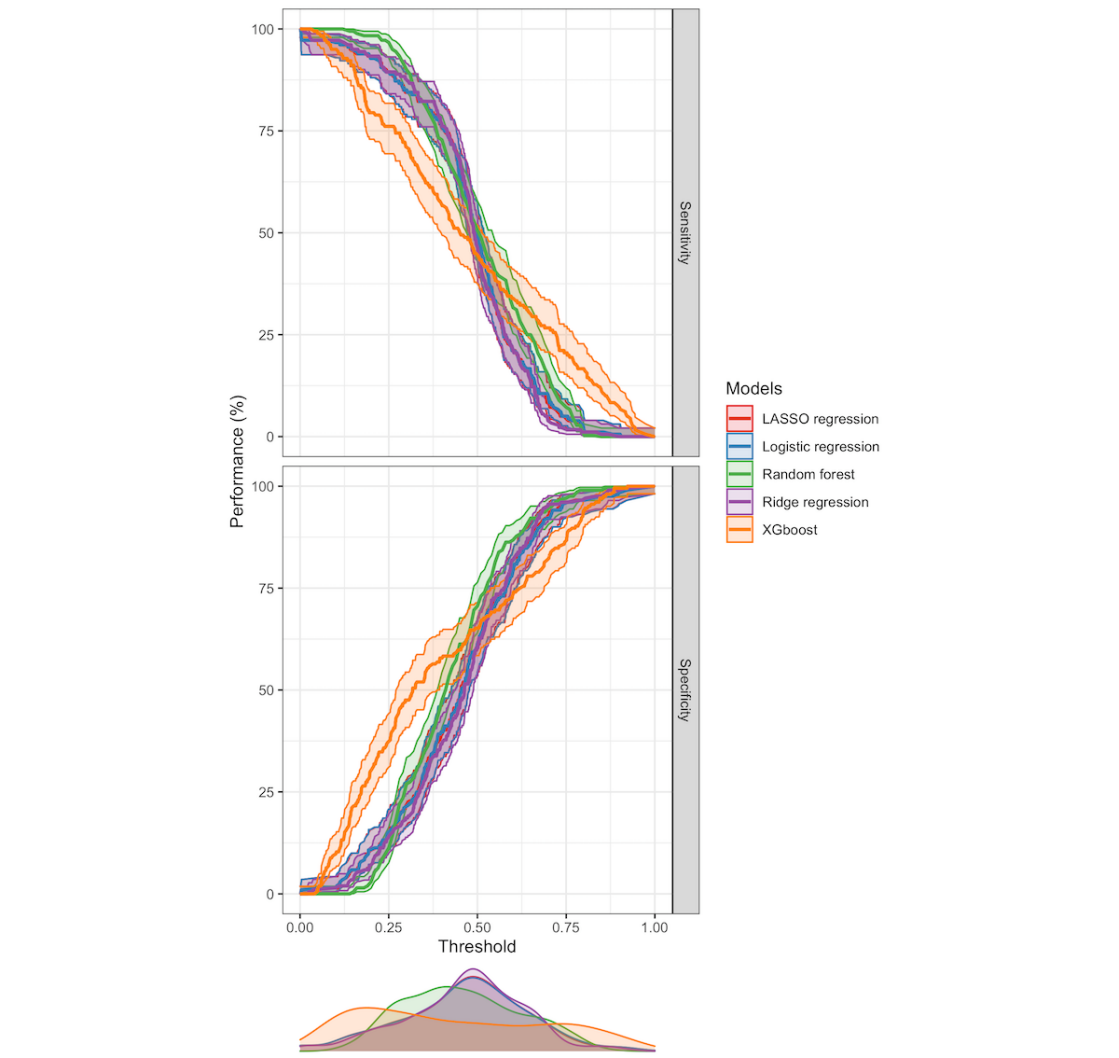
Threshold performance plot for all models evaluated. LASSO, least absolute shrinkage and selection operator; PPV, positive predictive value; NPV, negative predictive value.

A plot of the AUROC for the models using predictions from the 10-fold cross-validation is presented in Figure 1. The random forest model had the best discriminatory power of the models, with a mean AUROC score of 0.66 (SE 0.03). A threshold performance plot, which summarizes the discriminatory power for the models, including values for sensitivity, specificity, positive predictive value, and negative predictive value across all probability thresholds, is presented in Figure 2.

### Calibration

**Figure 3:**
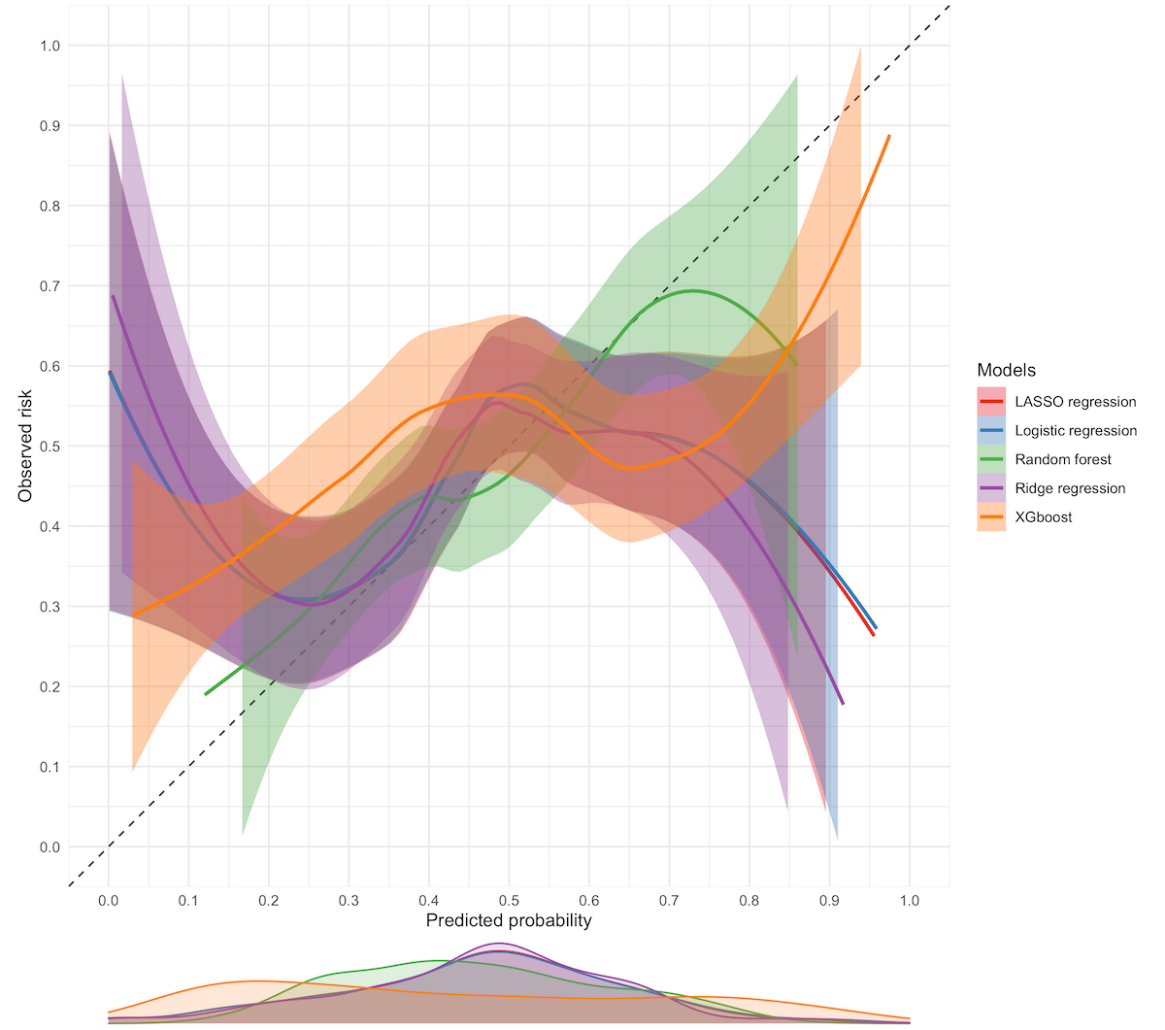
Calibration plot for all models evaluated. LASSO, least absolute shrinkage and selection operator.

The random forest model had the best calibration. It approximated observed risk at moderate (0.5) to high (0.8) thresholds (Figure 3), although the risk was overestimated at very low thresholds and slightly underestimated between 0.4 and 0.5. Other models severely overestimated risk at low probability thresholds and underestimated risk at high probability thresholds.

### Decision Curve Analysis

**Figure 4:**
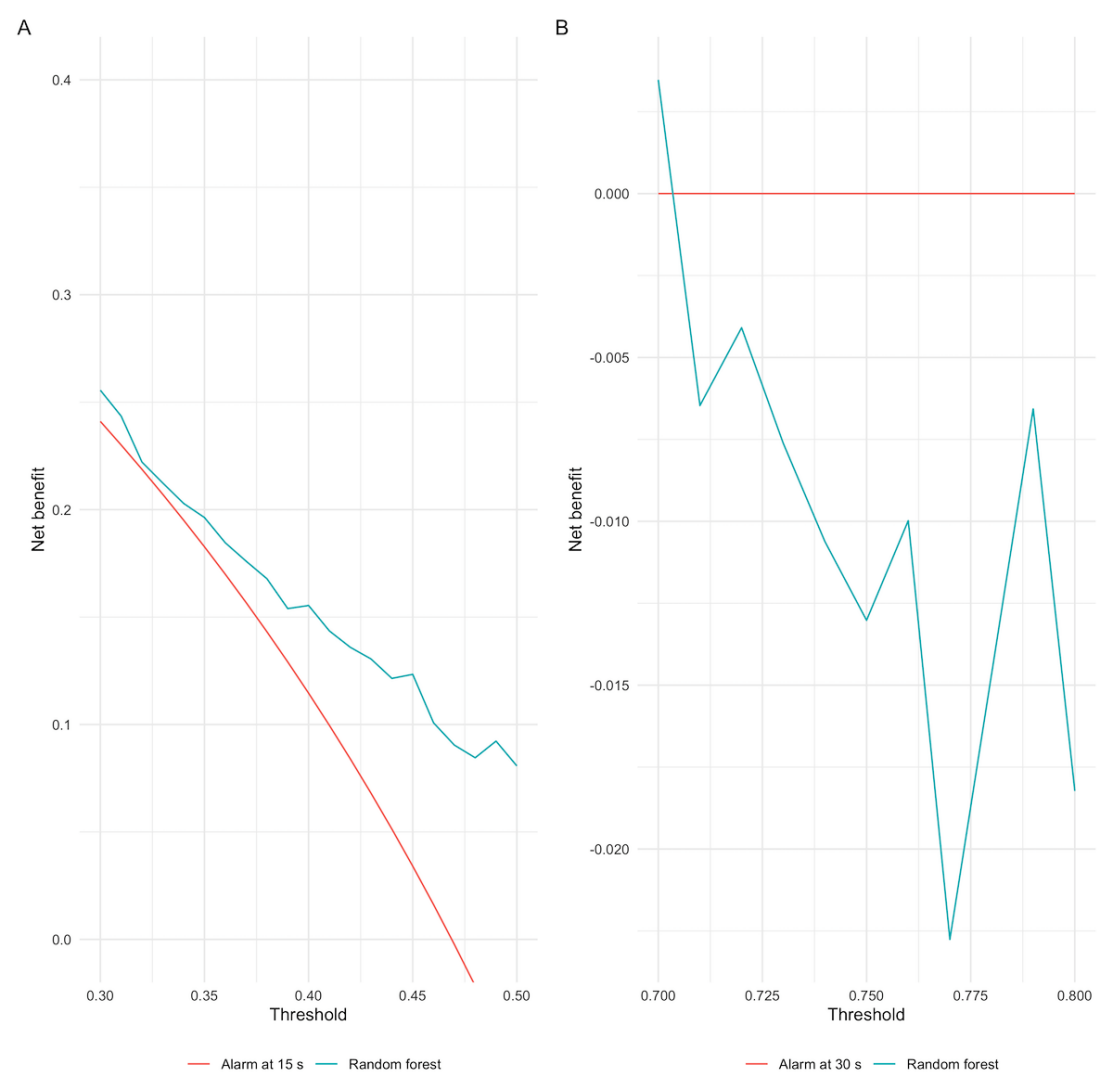
Decision curve analysis plots.

As the random forest model performed the best in terms of discrimination and calibration, we chose this model for evaluation using decision curve analysis. The net benefit associated with the random forest model exceeded that associated with the aggressive strategy across all probability thresholds in the range of 0.3-0.5 (Figure 4). The interpretation is that the best clinical outcome would be achieved for clinicians who are willing to initiate intervention for apnea at the 15-second mark if the probability of the event being prolonged was more than 40% by using the random forest model. The net benefit associated with the random forest model was lower than that associated with the conservative strategy across all probability thresholds in the range of 0.7-0.8 (Figure 4). Figure 4A is the comparison of the model with the aggressive strategy and Figure 4B is the comparison of the model with the conservative strategy.

## Discussion

In this study, we found that the random forest model had the best discriminative ability and calibration for predicting if an apneic event would be prolonged during nurse-administered procedural sedation. However, it should be noted that the accuracy of this random forest model was still quite low (AUROC 0.66). Additional research is needed with larger sample sizes to validate our initially promising findings.

Results from prior studies indicated that the use of information about the history of previous respiratory states may be a promising approach for predicting the duration of apneic events. A study of capnography waveform abnormalities during nurse-administered sedation found a two-fold increase in the risk of apnea (hazard ratio [HR] 2.14; 95% CI 1.75-2.62) when a patient was in a state of hypoventilation (defined as >10% reduction in end-tidal CO_2_ from baseline) [24]. The risk of apnea also increased with each additional sedative dose (HR 2.86; 95% CI 2.15-3.81) [24]. Results from an earlier study in a different population also supported the observations that the onset of apneic periods during sedation is associated with a previous history of abnormal respiratory state. Krauss and colleagues [25] used survival analysis to model the time to first apneic events in a sample of 312 patients undergoing procedural sedation with propofol or ketamine in the emergency department. They found that the risk of apnea increased with an abnormal end-tidal CO_2_ measurement 30 seconds (HR 2.45; 95% CI 1.63-3.69), 60 seconds (HR 1.88; 95% CI 1.21-2.92), and 90 seconds (HR 2.06; 95% CI 1.36-3.11) prior to an apneic event. In our study, we leveraged information about the associations between apneic events and the history of previous respiratory states by building a predictive model using a machine learning approach. Features included in the models we tested were the previous respiratory state, duration of time in the previous respiratory state, number of previous apneic events, and duration of the previous apneic event.

Many prediction modeling studies focused on predicting clinical outcomes have yielded similarly low AUROC scores. For example, a recent study of the predictive ability of vital sign parameters for clinical deterioration in subacute care patients reported an AUROC score of 0.57 [26]. Decision curve analysis can help elicit whether a model with low AUROC scores is “good enough” to use in practice. Our results indicated that nurses currently using the conservative strategy who are willing to value a false positive about 2-3 times more than a true positive would not derive an overall net benefit from using the random forest model as a trigger for apnea alarms. This is because the random forest model would produce a worse outcome than the default strategy of waiting for an alarm to be triggered at the 30-second threshold in terms of the balance between true positives and false positives for determining if an apneic event will be prolonged.

Conversely, nurses currently using the aggressive strategy who are willing to value a false positive about 2-3 times more than a true positive would derive an overall net benefit from using the random forest model as a trigger for apnea alarms. Using the random forest model as an additional input for an alarm trigger would reduce the total alarm burden and could be considered an option for implementation into practice. To operationalize these predictions into capnography monitors, partnerships with industry would be required because monitor functionality would need to be adapted to facilitate input of the data required to calculate the predictions [27]. These data would include patient characteristics and sedative dosing. Integrating predictive models into alarm management strategies for respiratory monitoring devices is also indicated in other contexts. For example, a recent study found that opioid-induced respiratory depression during recovery from anesthesia can be accurately predicted using a machine learning approach [28]. In addition, user-centered design considerations, such as how the predictions should be communicated to nurses responsible for decision-making, are important avenues for further research prior to implementation [29].

This study used decision curve analysis to evaluate the potential clinical impact that using the model as input for capnography alarm management would have on the number of alarms triggered (ie, false positives and false negatives). However, as with any intervention in health care, the efficacy of the model needs to be assessed prior to broader implementation. The indicator for efficacy in this context would be the improvement in patient safety using this model as input for capnography alarm management. The gold standard approach for such an evaluation is a randomized controlled trial. Randomized controlled trials testing alarm conditions that have integrated predictions from machine learning models have been conducted previously in similar contexts such as intraoperative blood pressure management [30,31].

A noteworthy finding is that the model produced an overall net benefit that was higher than that of the aggressive strategy but not higher than that of the conservative strategy. Further research with larger sample sizes is needed to increase the predictive power of models aimed at predicting the duration of apneic events. Such research is warranted because triggering an alarm after 30 seconds of apnea that would turn off without clinical intervention only 5 seconds later is just as clinically inconsequential as triggering an alert after 5 seconds of apnea that would similarly turn off after a short time. In both these circumstances, there would not be enough time for the clinician’s intervention to take effect. However, presumably in an attempt to reduce alarm burden, the default settings for many capnography monitoring devices are for the alarm to be triggered after 30 seconds of apnea. An ideal alternative to the conservative strategy would be for capnography monitor alarms to be triggered as early as possible during an apneic event, but only if the event will be prolonged enough to necessitate clinical intervention and for this intervention to take effect—a goal that we did not achieve in this study. However, previous research indicates that it would be worthwhile to find such a solution. An analysis of half a million patients found that respiratory compromise during interventional radiology procedures performed with moderate sedation led to worse clinical outcomes and higher costs than those observed in normal respiratory states [32].

### Limitations

Although the number of apneic events included in the models was relatively high, this was seen in a small number of patients. We used cross-validation to minimize the possibility of overfitting. This analysis used data from an observational study conducted at two hospitals that used a convenience sampling approach; therefore, selection bias was possible. The context in which the study was conducted should also be considered in terms of external validity. Participants were patients undergoing procedures in a cardiac catheterization laboratory where small bolus doses of midazolam and fentanyl were used for sedation. Other procedural sedation contexts may involve the use of different sedative doses and types of medications, so the results of this study might not be generalizable to these contexts. A further limitation is that clinicians were not blinded to capnography measurements because of the observational nature of the study design. It is possible that interventions used by clinicians during the 0- to 30-second apneic period influenced the duration of the apneic event. However, this mimics real-world practice in that interventions may be implemented at clinicians’ discretion where no alarm conditions have been met. Additionally, 25% of the study population had sleep apnea, which was one of the predictors included in the model. Due to the small sample size, the dataset used to train the model would have contained only a small proportion of patients with sleep apnea and therefore it may not be generalizable to the larger population of individuals with sleep apnea. Further research with larger sample sizes is required to confirm our findings.

### Conclusion

We evaluated several candidate models to determine their accuracy in predicting at the 15-second timepoint if an apneic event would prolong for >30 seconds. The random forest model performed the best in terms of discrimination and calibration. Decision curve analysis indicated that using the random forest model for capnography alarm management would lead to a better outcome than using an aggressive strategy in which alarms are triggered after 15 seconds of apnea. The model would not be superior to the conservative strategy in which alarms are only triggered after 30 seconds.

## Abbreviations

AUROC: area under the receiver operating characteristic curve
CO2: carbon dioxide
HR: hazard ratio
